# Problems of geometry, sampling, and scale in gridded biodiversity data, and proposed solutions

**DOI:** 10.1016/j.ecoinf.2026.104112

**Published:** 2026-11

**Authors:** Petr Keil, Friederike Wölke, Gabriel Ortega-Solís, Florencia Grattarola, François Leroy, Florian Jansen, Carmen Soria

**Affiliations:** a Faculty of Environmental Sciences, Czech University of Life Sciences in Prague, Kamýcká 129, Praha-Suchdol, 165 00, Czech Republic; b Department of Ecology, Environment and Plant Sciences, Stockholm University, Svante Arrhenius väg 20 A, Stockholm, 106 91, Sweden; c Department of Evolution, Ecology and Organismal Biology, The Ohio State University, Columbus, OH, 43210, USA; d Faculty of Agriculture, Civil and Environmental Engineering, University of Rostock, Justus-von-Liebig-Weg 6, Rostock, 18059, Germany

**Keywords:** Biogeography, Scale, Macroecology, Squares, Species-area, bias, Observation error, Imperfect detection

## Abstract

Grids, and gridded biodiversity data such as regional or country-level atlases, play a prominent role in ecology, particularly in the study of spatial patterns of species occupancy, geographic ranges, biodiversity, and their drivers and temporal dynamics. However, managing, exploring, and analyzing gridded data presents numerous challenges. Here, we review the problems with gridded data, and the existing solutions. We hope to provide guidance, particularly to early-career ecologists who may otherwise struggle to make sense of the various solutions scattered throughout the literature. We focus on grid-specific problems of sampling (e.g. varying sampling method and effort in space and time, imperfect detection), geometry (e.g. varying grid cell area and shape, positional errors), and scale (e.g. spatial grain and temporal extent). Solutions can be implemented before gridding, during data processing, or during data analysis. These include selecting appropriate projections and grid designs, aggregating or filtering grid cells, and accounting for biases within statistical models. By exposing gaps, trade-offs and single solutions to multiple problems across existing approaches, we further aim to inspire the development of novel modelling frameworks that tackle multiple challenges simultaneously.

## Highlights

• Fourteen recurrent challenges affecting gridded biodiversity analyses are synthesized.
• Challenges are classified into geometry, sampling, and scale domains.
• Sampling biases are most common and have multiple solutions.
• Temporal-scale challenges are the least known and hardest to overcome.
• Grid coarsening and hierarchical models address several problems simultaneously.

## 1 Introduction

In biogeography, macroecology, and applied ecology, data on species distributions are often arranged in a grid (Fig. 1, Box 1). Prominent examples are gridded atlases (Asher, 2001; Keller et al., 2020; Preston, 2013; Šťastný et al., 2021) (Table 1). Compared to local and spatially discrete data from local observations as in most data in GBIF (www.gbif.org) or in USGS North American Breeding Bird Survey (Ziolkowski et al., 2022), grids have the advantage that their cells are directly adjacent. This enables mapping the data with no blank spots, and it also ensures a thorough, continuous coverage of environments—useful when, for instance, modelling distributions of species that are absent in some suitable locations (Svenning and Skov, 2004), or when assessing occupancy and species ranges (Warren et al., 2001). Further, because grids are contiguous, we can assess not only occupancy, but also spatial patterns such as aggregation or fractality (Gaston, 2003). Importantly, adjacent cells can be aggregated to create coarser cells (Hurlbert and Jetz, 2007; Rahbek, 2005) or can be analyzed using moving windows (Lennon et al., 2001). This is necessary for analyses of scale-dependent phenomena such as species-area relationships (Storch, 2016), effects of abiotic environment on species-environment associations (Belmaker and Jetz, 2011), or occupancy-area relationship (Kunin, 1998), including their change in time (Leroy et al., 2024). Gridded biodiversity data are thus a vital ecological resource.

**Fig. 1:**
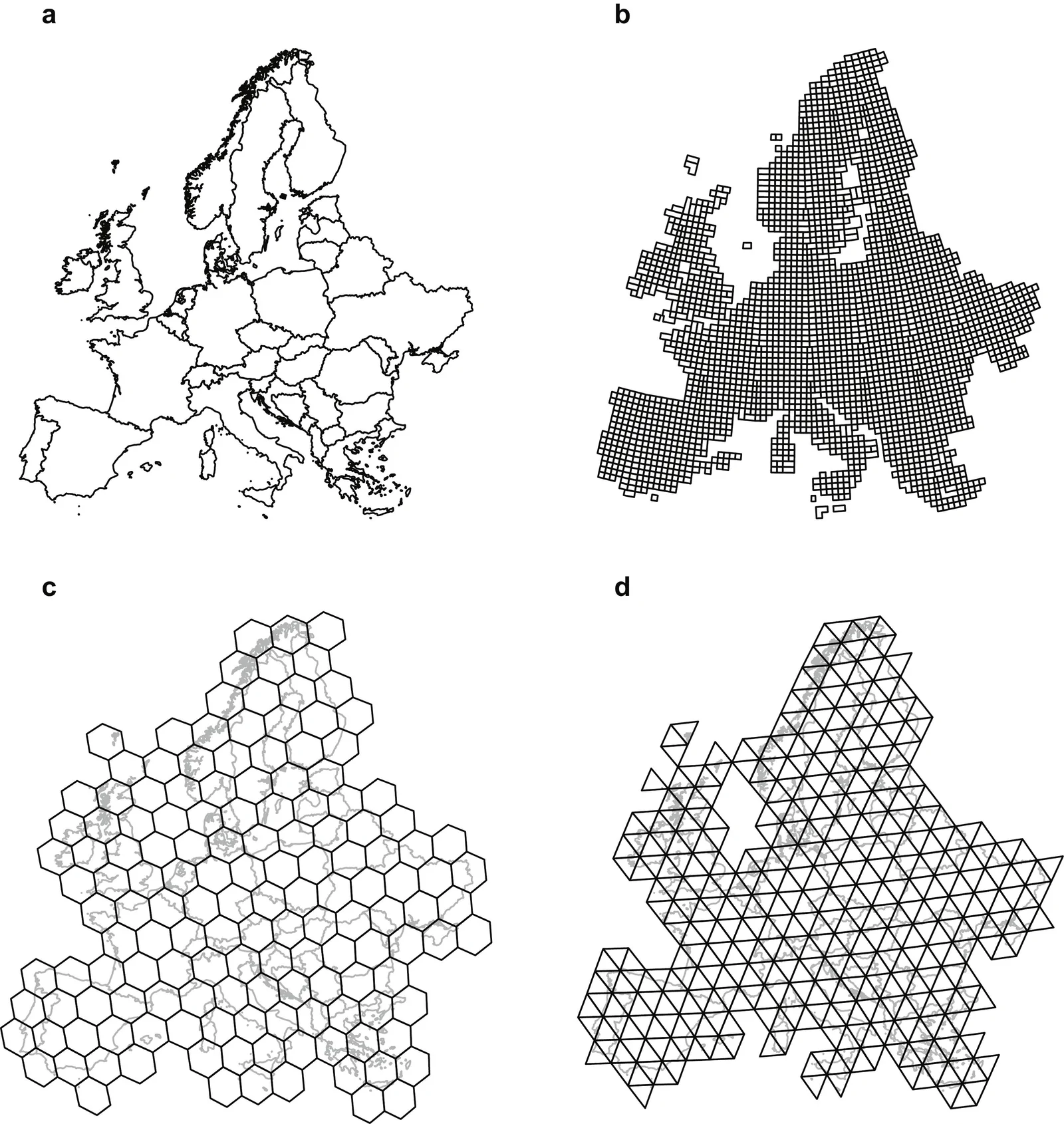
Examples of spatially contiguous grids. (a) A grid where cells are European countries, and they vary considerably in their area and shape. (b) A rectangular UTM grid used in Keller et al. (2020); note the irregular non-square shapes of some cells. (c, d) Hexagonal and triangular grids; their cells are only minimally distorted by geographic projections. Note that we are not dealing with grids of spatially discrete points in this review.

Box 1 **Basic definitions**

**Table:** 

Here we use the term **grid** (also called a mesh) for a structure consisting of multiple grid **cells** (polygons) of non-zero area, arranged in a **contiguous** way so that each grid cell is adjacent to another (Fig. 1); we thus leave out point meshes. In our view, grid cells may have varying area and shape even within one grid, and thus we also consider a set of adjacent countries (Essl et al., 2013) or a set of smaller adjacent administrative units (Craven et al., 2020) to be grids. **Gridded biodiversity data** describe the distribution of one or more species, or their diversity, across the grid. A typical example is a gridded **atlas** (Table 1) in which data values are always defined per grid cell. These data can be detections or non-detections of species, their presence and/or absences, some metrics or proxies for abundance, species richness, or other indices of diversity. The *average* area of the cell is the grid's **spatial grain** (resolution), and the distance or area between the grid's outer boundaries is the grid's **spatial extent**. For some ecological quantities (biodiversity and related concepts such as beta diversity, species associations, or species composition), there is no “correct” or “best” grain of a grid, and fine-grain grids are not better than coarse ones, they simply carry different information (Rahbek, 2005). This may come as a surprise to researchers from remote sensing, where the finer the raster resolution, the better. Finally, a **grid origin** is the exact geographic location of a selected fixed point of the grid; if we keep the grain and extent constant, but we change the origin, we are effectively moving the grid around the map. **Structured vs semi-structured vs unstructured gridded data** (Table 1). The highest-quality gridded data derive from **structured** surveys with standardized protocols that pre-specify the sampling method, effort, site selection, and temporal replication. **Semi-structured** datasets also derive from surveys that partly standardize sampling protocols and site selection, but they allow some flexibility in the number of observers, the time they spend in the field, the number of visits, and the selection of sites. Importantly, this variation is usually documented in metadata associated with the data collection. Finally, gridded data can be created through **unstructured** sampling processes, such as opportunistic observations, by overlaying point observations on a grid. In this case, the observation process is unknown, and there is little to no metadata on the biases inherent in the data collection. Many museum collections and most citizen science initiatives (e.g., eBird, iNaturalist, Observation.org) gather unstructured point occurrences (Pocock et al., 2017) widely available through GBIF.

**Table 1:** Examples of gridded atlas datasets, ordered from most structured (top rows) to least structured (bottom rows). Note that the Japanese bird atlas also includes additional unstructured data, which can be, however, filtered out.

Dataset	Type	Spatial extent	Spatial grain	Temporal extent	Sampling effort	Effort units per cell
Birds of Japan (Ueta et al., 2023)	Structured	Japan	20 × 20 km^2^	2016–2021	Known, fixed	
Czech bird atlas (Šťastný et al., 2021)	Semi-structured	Czech Republic	11.2 × 12 km^2^	2014–2017	Known but varies	# cards, # observers
SABAP2 - South African bird atlas (Brooks et al., 2022)	Semi-structured	South African countries	5′ × 5’	2007-present	Known but varies	# cards, hours
Mammals of Hamburg (Schäfers et al., 2016)	Unstructured	Hamburg, Germany	2 × 2 km^2^	1997–2015	No	

Managing, exploring, and analyzing data on species distributions in a grid, however, comes with problems. Perhaps the most discussed in the literature is the problem of incomplete or biased sampling (Boyd et al., 2021, Boyd et al., 2024; Isaac et al., 2014; Rapacciuolo et al., 2021). Less discussed are problems with geometry (Kunin, 1997) and scale (Boyd et al., 2024; Hurlbert and Jetz, 2007). Furthermore, there may be problems, decisions, and solutions that are not part of methodological overviews, but are described in specific biodiversity analyses in methods sections, appendices, protocols, and workflows. These may be scattered, and motivations for decisions are not always well described or justified: we may see what people did, but we don't know why.

In this paper, we review problems associated with gridded data, summarize their causes, and propose solutions. We focus specifically on geometry, sampling, and scale, the areas closest to our expertise. The proposed solutions aim to (i) make raw gridded data more informative in visualizations and exploratory analyses and (ii) make these data more suitable for statistical modelling. Rather than offering a single solution to every problem, we present a toolbox of approaches and show that some address multiple problems simultaneously. By identifying connections among the problems, we expose gaps in current analytical approaches and highlight opportunities for improvement. The problems and solutions apply both to existing grids and gridded atlases and to the design of new grids. Ultimately, our goal is to support accurate maps and adequate models of species distributions and diversity that represent reality and provide reliable predictions and inferences.

## 2 Literature processing

To identify literature on gridded biodiversity data, we combined the following approaches. (1) In 2023, we searched Web of Science and Scopus for the string “atlas AND biodiversity,” which yielded 351 papers. (2) We used the AI tool Elicit to scan these papers and extract information about taxa, temporal replication, data type, geographic extent, spatial scale, and metrics. After reviewing the output, we read all relevant papers individually, examined their cited references, and assigned specific problems and solutions to individual co-authors for further investigation. However, we did not use generative AI to write the manuscript; we wrote it manually. (3) We added 59 papers with which we were already familiar through our extensive experience with the subject, as well as additional relevant papers suggested by colleagues who work with gridded atlases, specifically Nick Isaac, Diana Bowler, and Oliver L. Pescott.

## 3 Geometrical problems

Problems of geometry affect almost every grid to some degree. These problems include variation of grid cell shape, or variation of the shape of the habitable area within cells (Fig. 2). Many of these are caused by geographic projections. For example, in rectangular grids with global extent and equal-area projections, there is increasing north-south elongation of cells towards the poles. An example is the Behrmann projection of global vertebrate grids (Coelho et al., 2023; Faurby et al., 2018), or the European 50 × 50 km^2^ UTM grid (European bird census council, 2020) with increasing fraction of small grid cells northwards. Second, cells may have an irregular shape by definition; these can be countries, biogeographic regions, or counties (e.g. Craven et al., 2020). Finally, problems of geometry often arise when grids are created by aggregation (“coarsening”) of other grids, when coarse-grain grid cells are not fully filled by fine-grain cells, particularly around the grid's outer boundary (Keil et al., 2011).

**Fig. 2:**
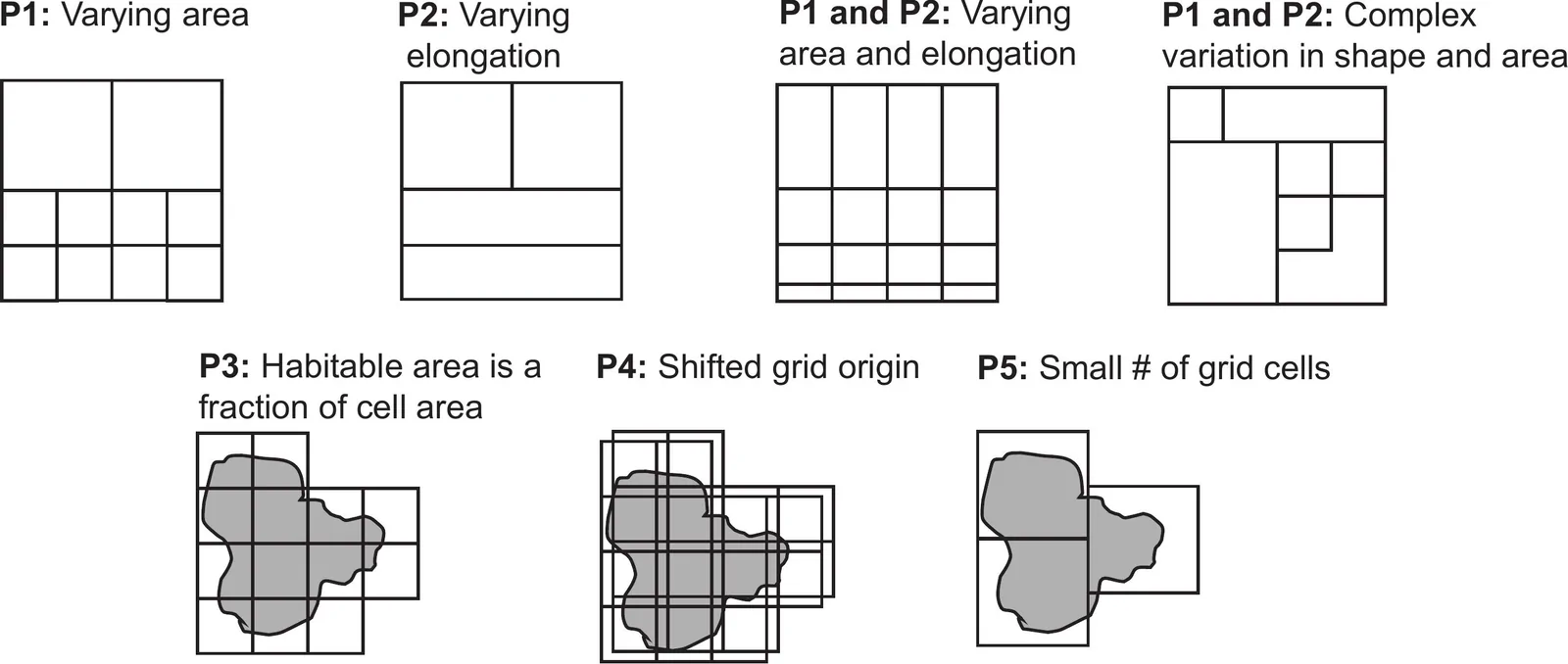
Common geometrical problems of gridded biodiversity data.

### 3.1 Overview of geometrical problems

**P1: Varying cell area.** Some cells in a grid can be larger than others (Fig. 2). This severely affects prevalence (occupancy) of every species, since it follows a positive occupancy-area relationship (Kunin, 1998); in other words, the larger the cell, the larger the probability that a species is in it. Consequently, variation of cell area can mask, or distort, geographic patterns and other drivers of distributions. The same problem applies to species diversity, which follows the species-area relationship (Arrhenius, 1921; Storch, 2016): the larger the grid cell, the more species are in it. Furthermore, variation in cell area distorts beta diversity, species co-occurrences, and species composition in grids, since they are all derived from species distributions and/or from diversity patterns.

**P2: Varying cell elongation.** Some cells can be elongated more than others (Fig. 2). The more elongated the shape, the larger the variety of environments in the cell, and the more species in the cell. This is because of Tobler's law which states that “everything is related to everything else, but near things are more related than distant things” (Tobler, 1970). The more elongated cells are, the longer gradients of environments and species composition they cover, and species compositional similarity decays with distance (Nekola and White, 1999). This decay is due to varying environmental niches among species, but can also emerge from purely neutral processes in homogeneous environments (Rosindell et al., 2011). Consequently, given constant grid cell area, more elongated grid cells have higher per-species probability of occupancy and more species than square or circular cells (Kunin, 1997), affecting patterns of species occupancy, diversity, and beta diversity. Thus, when elongation of cells varies, it can distort several biodiversity patterns independently of area.

**P3: Habitable area is a fraction of cell area.** The world is full of boundaries and barriers that delineate habitable areas for species (Fig. 2). An example is the coastline delimiting land for terrestrial species. When we overlay a grid cell over the boundary, we end up with only a fraction of the cell covered by the habitable area; for example, in the grid of the European bird atlas (Keller et al., 2020) the only land in a ~ 2500 km^2^ cell is Ibiza, an island of 573 km^2^, which is 22.9% of the cell area. Because of this small inhabitable area, the number of terrestrial species in the cell is likely lower than in an inland cell of continental Spain with 100% of mainland. Consequently, even in a regular grid with constant cell area and shape, variation of the fraction of habitable area may distort patterns of species occupancy, distributions, and diversity. Indeed, sharp habitat or biome boundaries may cause the same problem.

**P4: Shifted grid origin.** When we shift the whole grid (we move the origin of the grid, Fig. 2) by less than a grid cell length, we affect occupancy and apparent distribution of species (Witte et al., 2008), and consequently also patterns of diversity. This problem emerges during creation of new grids, or in already existing older and newer grids in the same region, e.g. in the Czech atlases (Šťastný et al., 2021). The problem of shifted origin gets more serious the coarser the grain of the grid (Witte et al., 2008) because (i) there are fewer cells, and thus smaller sample size for statistical analyses, and (ii) because rare species located close to cell boundaries can completely reverse the spatial pattern.

**P5: Small number of grid cells,** e.g. at coarse resolutions or small extents. Some grids have a small number of grid cells natively, others because they have been created by aggregation (coarsening) of finer grids (Craven et al., 2020; Rahbek, 2005). If the number of cells becomes too low, it may compromise statistical estimators that need sufficient sample size.

### 3.2 Solutions to geometrical problems

Some of the solutions (S1–2 and partly S5) are only applicable when we have the luxury of designing the grid, which can either be when a new atlas initiative is launched, or before unstructured point observations (e.g. from GBIF) are aggregated to a grid. Other solutions (S3-S8) are more practical as they also work for already existing grids.

**S1: Use equal-area projection.** A popular way to avoid varying area caused by geographic projections is to use a regular grid in an equal-area projection; these projections can be custom tailored for any region using projectionwizard.org. However, note that, for rectangular grids, equal-area grids cause variation in elongation, which also affects diversity and distributions (Kunin, 1997); see also P2. This problem grows with increasing geographic extent of the grid, with global grids being most affected. A typical example is the popular Behrman equal-area projection of global grids (Faurby et al., 2018; Jetz et al., 2012), with extremely elongated cells at high latitudes. Thus, for these large spatial extents, hexagonal grids may be preferable (S2).

**S2: Use triangular or hexagonal grids.** Triangular and hexagonal grids don't require special projections, and they keep area, distance between cell centroids, and shape constant. In other words, they can be wrapped around a sphere while avoiding shape distortion. An example use of a global hexagonal grid is in Keil and Chase (2019). The disadvantage of hexagons is that they look unfamiliar, they cannot be easily converted to rasters to be processed using fast raster-based algorithms, and are challenging to be made nested, i.e. aggregated to coarser resolutions; but see Jurasinski and Beierkuhnlein (2006). Triangular grids can be coarsened and made nested, but we are unaware of any use of a triangular grid in biogeography.

**S3: Record the area and shape for each cell.** If the previous solutions can't be applied (e.g., because the grid is given and we have no control over its geometry), or when the habitable area is just a fraction of grid cell area, then the geometrical problems should be quantified and noted. We suggest that, for each cell in the grid, metadata are provided that specify: cell area, fraction of habitable area within the cell (e.g. fraction of mainland if terrestrial organisms are mapped), fraction of cell area covered by smaller grid cells (if the grid is an aggregate of a finer grid), and some metrics of shape deformation, e.g. circularity, linearity, or elongation ratio (Stojmenovic and Nayak, 2008). These metrics can then be used as covariates in statistical models (S7), such as in Leroy et al. (2024), or they can be used to filter out problematic cells that don't meet certain criteria (S4).

**S4: Remove cells with undesirable geometry.** These can be cells with excessive shape distortion (too elongated), cells with a small fraction of land, or cells that are too small or too large. Examples of this are cells overlapping coastal areas or the thin cells—sometimes less than 1 km wide but 10 km long—created when two or more contiguous UTM zones are used to build a grid. A simple and brute-force solution is to remove such cells or merge them with their neighbors, which usually requires a threshold to be specified, for example a threshold of a minimum area of land within a grid cell. Threshold values for this removal can vary in strictness, e.g. only include cells with 30% or with 100% of land (Keil et al., 2012), and the decision about the threshold is often arbitrary or intuitive, and there will likely be a tradeoff between sample size (number of cells) and the threshold strictness. Furthermore, removing cells with small amount of land mass may lead to disproportionate exclusion of coastal grid cells, which may be undesirable when coastal specialist species are of interest, or when the grid covers an island region with complex coastline such as in Japan (Ueta et al., 2023) or New Zealand (Bull et al., 1985) atlases. In these cases, recording the area and shape of landmass and using it as a covariate in models (see S7 below) is preferable to cell removal.

**S5: Test sensitivity of patterns to grid origin.** This can be wise particularly for coarse grids with few grid cells. When a pattern changes considerably with grid origin, avoid general conclusions from models based on these grids. When a fine-grain grid of the same extent as the coarse-grain grid is available, we can use moving windows (Lennon et al., 2001), i.e. a large sampling cell is moved continuously over the fine grain grid, and values such as diversity are calculated per fine-grain cell in the center of the sampling cell. This is equivalent to overlaying many coarse-grain grids with different origins. However, we should stress that biodiversity patterns specific to a particular grid origin are not incorrect, even when different from patterns observed in other grid origins. A particular grid origin may even be practical and preferable, e.g. when it leads to more even geometry of habitable area within cells, or when it matches a certain administrative division of a region.

**S6: Coarsen up the grid.** Some problems of varying cell area and shape can be fixed by aggregation of smaller cells to larger units, as done for example by Craven et al. (2020). This can be also done to ameliorate problems of positional errors (Moudrý and Šímová, 2012), although there is some controversy around that (Gábor et al., 2022). The universal downside of coarsening up the grid (see also S14 and S21) is reducing geographical detail and sample size; however, sometimes this may be a price worth paying for the increased reliability of diversity and distributions at coarse resolutions.

**S7: Use area and shape in statistical models.** When cells vary in their *area* (both, the cells themselves, and the proportion of the cell covered by habitable land), there are ways to include the effect of area in statistical models. First, a mathematical transformation of area values may be necessary and we strongly recommend it, usually log- or square root transformations. Second, the transformed area can be used as a predictor in models of diversity and distributions, ideally also as an interaction term with other predictors, e.g. see Keil and Chase (2019) and also S24. The latter stems from the well-established observation that sample area not only affects diversity and distributions per se (Storch et al., 2007), but it also modulates the effects of other variables (Belmaker and Jetz, 2011). Furthermore, some types of hierarchical statistical models assume a very specific effect of area so that it is used in models as an offset (Grattarola et al., 2023, Grattarola et al., 2024; Isaac et al., 2019). Regarding the *shape* attributes, a common problem is varying elongation, which is common in both rectangular and irregular grids. Compared to area, there is much less research on how (and if) elongation should be transformed, and how it interacts with other predictors. Here, we recommend standard model selection procedures to assess the effect of elongation, as well as investigating the statistical interaction between shape and area.

**S8: Avoid generalizations from a few cells.** When the number of cells is small, for example at very large grains, it may still be useful to calculate some quantities per grid cell; for example, we may be interested in the total number of species (species pool) in a large region corresponding to a large grid cell. However, statistical generalizations based on these small samples are not advisable. If generality is desirable, use a finer grid with more grid cells. Alternatively, Bayesian statistics can quantify the uncertainty caused by small sample size (Gelman et al., 2013).

## 4 Sampling problems

Sampling problems (Fig. 3), specifically those related to variation in sampling effort and methods, are perhaps the most common and influential problems in gridded biodiversity data. They were also a subject of several reviews (Botts et al., 2012; Boyd et al., 2023; Isaac et al., 2014; Robertson et al., 2010). The ultimate reason for sampling problems is that we mostly cannot survey an entire grid cell, nor can we survey it continuously over time. Instead, we are left with spatially and temporally discrete samples (plots, point counts, surveys, visits) or incidental records. The total number of such samples per grid cell, combined with the total number of individuals per sample, sample area or length (when the sample is a transect), or time spent sampling, represent *sampling effort*, and the lack of information on sampling effort is behind most of the problems listed in this section.

**Fig. 3:**
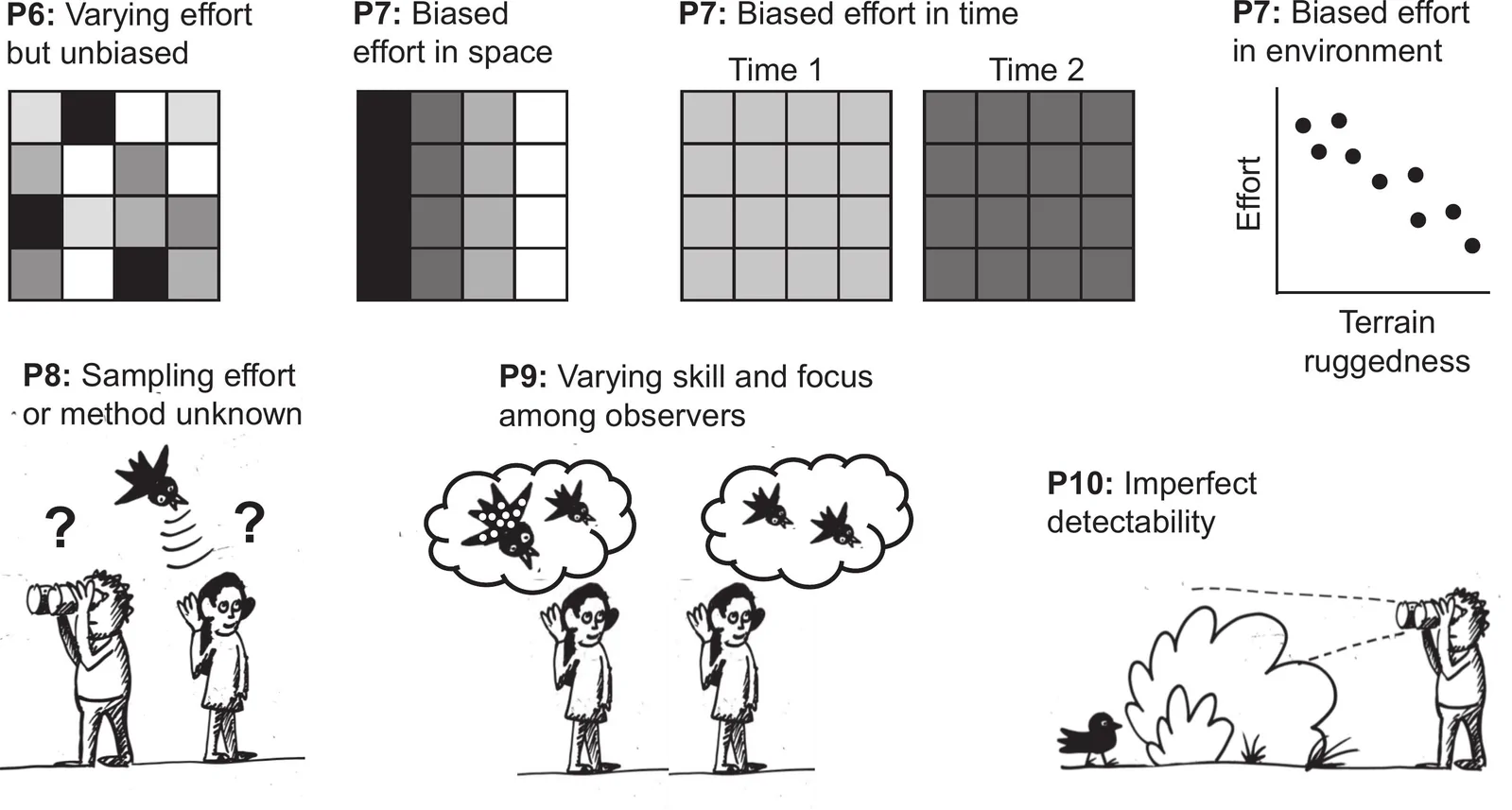
Common sampling problems of gridded biodiversity data.

### 4.1 Overview of sampling problems

**P6: Varying sampling effort.** Sampling effort can vary geographically, temporally, or spatiotemporally, which can render some grid cells in certain periods under-sampled. This may affect some ecological quantities, e.g. occupancy of species, average richness, or regional richness. However, if the variation is random in space (Fig. 3), time, or environment, it may not be such a problem for geographic, temporal, or environmental gradients of diversity and distributions. However, this randomness will likely be rare.

**P7: Biased sampling effort in space, time, or environment.** This is a special case of the abovementioned problem, when sampling not only varies, but is also systematically biased (clumped) in space (Fig. 3), time (Fig. 3), or both, for instance when some regions are better accessible or attractive. Temporal biases (Bowler et al., 2022) (Fig. 3) can emerge when the weather (or climate) in certain time periods is better for sampling, or with the general increase in biodiversity records towards the present (https://www.gbif.org). This issue might also arise due to a shift in sampling focus from one area to another, e.g., when one region is already well-sampled, and the surveyors move to another region. Even worse, there can be a joint bias both in time and space, with some regions sampled only in a certain period. This can be coupled with biases in environmental space (Xu et al., 2024), e.g. when some environments are less accessible or attractive to observers (Fig. 3). All these biases lead to detected patterns of diversity and distributions that differ from real-world patterns. In the most extreme, real-world patterns are then completely obscured or reversed by patterns of sampling (Grattarola et al., 2020).

**P8: Sampling effort or method unknown.** Sometimes we may have no information on the sampling effort. This can be further complicated when sampling methods are unknown, or insufficiently described. In such cases it may seem impossible to determine if non-detections of species are real absences (Dobson et al., 2020; Rapacciuolo et al., 2021).

**P9: Varying skill and focus among observers.** Ecological data collection usually involves many observers with various levels of expertise, impacting data quality (Isaac et al., 2014). Even when considering systematic data collection, less-skilled observers may work slowly, are more likely to miss cryptic species, and tend to mis-identify species. Consequently, even if sampling effort in observer hours per grid cell is constant, the real sampling may vary, i.e. it is lower in cells surveyed by unskilled observers (Prendergast et al., 1993). Since identification relies on visual and auditory cues, the ability of observers to detect the species around has been shown to decay with age (Farmer et al., 2014). Apart from skill, the focus may vary between observers, some preferring species from a certain habitat or certain type of taxa, e.g. some observers may be biased against alien species, or towards conspicuous taxa (Bowler et al., 2022; Johnston et al., 2023).

**P10: Imperfect detectability.** Detecting all individuals in a grid cell is not guaranteed due to combination of imperfect detectability and insufficient sampling effort, resulting in false negatives and inaccurate distributions (MacKenzie et al., 2002). The probability of detection at a grid cell depends on species abundance, conspicuity, and behavior, which are influenced by the cell characteristics (habitat, disturbance level), environmental conditions (including season, phenology, weather, time of the day), and survey method, timing, effort, and observer skill (Guillera-Arroita, 2017). As perfect surveys are unlikely, most raw gridded biodiversity data tend to have a lower number of detected presences than true occurrences of species.

### 4.2 Solutions to sampling problems

Solutions to sampling problems nearly always involve some estimation of sampling effort as the first step (S9-S12) and vary from adjustments of the data such as exclusion of under-sampled grid cells (S13) or coarsening up the grid (S14), to custom-tailored algorithms (S15-S16), and model-based solutions based on hierarchical models (S17-S20). The latter is both flexible and mathematically sound, but hierarchical models can also be challenging to specify correctly, can have high computational cost, and can come with steep learning curve. Thus, we encourage researchers to also consider simpler or more ad-hoc solutions, and/or to creatively combine them with hierarchical models.

**S9: Get data on sampling methods and effort.** We advise seeking data on sampling effort and methods in gridded biodiversity data whenever possible; if unavailable publicly, they may still be possible to get from the authors. Data on effort can take many forms, e.g. number of traps, time of trap exposure, number of transects and their length, or surveyed point locations. In semi-structured atlases (e.g. the Czech bird atlas; Ortega-Solís et al., 2026), proxies of effort can be the number of survey cards per cell (cards containing lists of species detected during a visit), number of observers per grid cell, or total number of observer hours over all visits in a cell. Sampling effort may be further standardized to a relative measure of sampling *completeness* (Keller et al., 2020). These metrics of effort can be then used in statistical models as covariates, as we describe below. Here we also caution that even though some of these data may be close to a real effort, they may not be the exact measure of it. For instance, just the number of observers per grid cell is not capturing the variation in hours per observer. Yet, even limited information on the reporting rate can improve models (Isaac et al., 2014).

**S10: Get data on recorder identity.** Knowing the identity of recorders can be used to determine *recorder syndromes* (Isaac and Pocock, 2015), i.e., typical sets of recording behaviors which can be estimated from species list lengths, coverage of rare or difficult-to-identify species, frequency of recording etc. These can then be used in statistical models that account for sampling variation (Isaac and Pocock, 2015) (S17, 18). However, recorder identity is sensitive personal information that is not usually freely available, not even in rare cases when it is known. Providing anonymized recorder IDs (e.g. recorder 547) could solve this issue.

**S11: Use external proxies of sampling effort.** In unstructured or semi-structured data, sampling effort often correlates with accessibility, e.g. modelled as distance to human settlements (Monsarrat et al., 2019) or distance to roads and research institutions (Meyer et al., 2015). Not commonly used but potentially useful may be the distance from attractive protected areas where observers enjoy working more than in cultural landscapes. These data can be extracted from public GIS layers and used as proxies for sampling effort in hierarchical models (S17, 18). The downside is that these proxies alone can only inform about relative effort values (e.g., better accessible grid cells are more likely to contain detections), but not about the absolute effort which would enable probabilistic statements about the actual probability of presence of the species.

**S12: Derive proxies of effort from detections.** Some methods use detections and non-detections of species to infer proxies of sampling effort. For instance, Szabo et al. (2010) proposed to use the number of species detected in a grid cell as an indicator of sampling effort; we see this method as questionable, since it confuses effort with one of the fundamental ecological quantities of interest. In a better alternative, Hill (2012) proposed to use the frequency of the so called “benchmark species” that are detected in the neighboring cells as an indicator of sampling effort. Encouragingly, we found that this frequency correlates well with some real metrics of sampling effort (under review). Hill (2012) then demonstrates how this can be used, in what is now known as the *Frescalo algorithm*, to infer species occupancy trends over time or to identify unexpected presences or absences. Studies that use the algorithm are Eichenberg et al. (2021) or Montràs-Janer et al. (2024). There is also a practical guide on how to use the algorithm (Goury et al., 2026). This proxy of effort can then be used in hierarchical models (S17-S20), although the same limitation applies as for the external proxies mentioned above: the Frescalo-derived proportion of benchmark species is a proxy of relative sampling effort, not absolute one (see S11).

**S13: Remove (filter out) under-sampled cells.** Once we have the sampling effort (S9), or its relative proxy (S11, 12), a crude but simple solution is to remove sites with sampling effort below a certain threshold. One way to determine the threshold is to model species richness as a function of the effort, and to determine where the function approximately flattens out (Prajzlerová et al., 2024). Indeed, removing cells means considerable data loss, particularly when assessing temporal change across multiple time periods (atlas editions), since even well-sampled cells in one atlas period may need to be removed if the same cells were under-sampled in another period. A good situation for this solution is when the total number of grid cells is high, e.g. at fine grains, and thus even exclusion of large number of under-sampled grid cells retains a large and representative sample of well-sampled cells. A good overview of filtering approaches is provided by Hughes et al. (2024).

**S14: Coarsen up the grid.** In macroecology, it has been recognized (Hurlbert and Jetz, 2007) that imperfectly sampled fine-scale data converge to true presences and absences if they are aggregated to sufficiently coarse grids. Coarser grids also give more accurate estimates of species occupancy, i.e. the proportion of occupied grid cells (Boyd et al., 2024). In other words, there is a minimum reliable grid resolution (Keil et al., 2014) above which we are safe to assume true presence and absence. This has been demonstrated to work for spatially random variation in sampling (Fig. 3) (Fig. S1 in Keil et al., 2014). This is also the reason why global biodiversity grids have grains of 100 × 100 km^2^ and coarser (Faurby et al., 2018; Orme et al., 2006) and why finer global grids (Jenkins et al., 2013) are problematic. As we also mention in S6, S8, S14 and S21, the downside of the increased reliability of coarser grids is the loss of detail and sample size.

**S15: Use rarefaction.** In a set of studies related through their data and authors (Biesmeijer et al., 2006; Carvalheiro et al., 2013; Keil et al., 2011), a variant of *individual-based rarefaction* (Gotelli and Colwell, 2001; Hsieh et al., 2016) was used to account for variation in sampling effort in gridded data, where records in each grid cell were down-sampled to a standard number to obtain standardized number of species per grid cell. Another example of the use of individual-based rarefaction over a country-wide grid is in Grattarola et al. (2020) or Klímová et al. (2025). It should also be possible to perform *sample-based rarefaction* (Gotelli and Colwell, 2001) to standardize effort across grid cells when biodiversity data are not just individual detections, but sampling events with lists of species per event (e.g. bird point count data or botanical plots). However, beware that we still lack a comprehensive validation of the rarefaction approach in the gridded large-scale setting either on simulated data, or on data with known true richness. Furthermore, the use of rarefaction is only justifiable when detections and/or samples within grid cells are not spatially too clumped, and/or distributed in an unrepresentative subset of environments in the grid cell.

**S16: Ad-hoc corrections of metrics of change.** Isaac et al. (2014) provide an overview of simple statistical tricks that have been proposed to control for varying effort directly in the calculation of metrics of temporal change. Examples of these include Telfer et al. (2002)’s estimation of change in species occupancy as a deviation from the overall occupancy trend of all species, or Ball et al. (2011)’s proposition to model species status in a cell as the proportion of total records in the grid cell. Although inspirational and potentially easy to implement, these are specific solutions linked to specific metrics and data types and should be used with caution.

**S17: Use sampling effort as a covariate.** An easy-to-apply method of controlling for sampling problems is to use additional data on sampling effort, method, or their proxies, in simple statistical models such as GLM or in machine learning. The formula of this approach is $Y=f\left(X,\mathrm{effort}\right)$, where Y is the ecological quantity of interest (e.g. occupancy or species richness) and X are predictors not related to sampling. This approach is valid, but it has a key limitation: It does not estimate the true occurrence of species (or species richness); it only estimates frequency of detections and richness of detected species, given a fixed value of sampling effort. We thus need to assume that frequency of detections corresponds to the actual species occurrence, which may not hold (MacKenzie et al., 2002). Further, the effect of effort on ecological quantities can be non-linear (Gotelli and Colwell, 2001) and effort also interacts in its effect with other variables, sometimes those affecting species detection (Kéry and Royle, 2015), making such simple statistical approach problematic.

**S18: Use hierarchical imperfect detection models.** There is a class of hierarchical models (Guillera-Arroita, 2017; MacKenzie et al., 2002) which separate the observation (detection) process from the true but unobserved ecological quantity such as occurrence or abundance of a species. These models can be used to account for varying effort and imperfect detection when estimating the probability of a species' presence. These models typically rely on repeated site visits, but these are not always available. One solution is to create “pseudo” visits by dividing unstructured or semi-structured records within an atlas period to shorter time intervals (Klinkovská et al., 2024), but for this we need to know the dates of the records. Another option is to use single-visit versions of occupancy models (Lele et al., 2012) which have been already applied to gridded atlas data (Peach et al., 2017; Sadoti et al., 2013); they either rely on external predictor of the observation process (it needs to be orthogonal to predictors of the ecological process), or on space-for-time substitution where adjacent grid cells are aggregated, which reduces the effective sampling size. Importantly, Pescott (2026) presents the connection between imperfect detection models and the Frescalo algorithm of Hill (2012), see also S12. The practical downside of hierarchical imperfect detection models used to be their steep learning curve and computational cost. However, this has become less of an issue lately, with convenient software packages such as spOccupancy (Doser et al., 2022) and spAbundance (Doser et al., 2024).

**S19: Leverage external data with integrated models.** Sometimes there can be other data types available for the region of the gridded atlas, and these can improve models that are based on the atlas data with varying sampling effort. Specifically, there are species distribution models which can calculate joint likelihood of multiple types of biodiversity data (Dorazio, 2014; Isaac et al., 2019), and we call these the *integrated models*. These have been used to integrate gridded records with local surveys to improve predictions of population trends (Pagel et al., 2014) or to integrate gridded records with expert range maps and camera traps to predict large-scale dynamics of geographic ranges (Grattarola et al., 2024). For an overview of the uses of integrated models in gridded citizen science data see Johnston et al. (2023). A convenient software package for integrated models is spOccupancy (Doser et al., 2022), although more complex data structures may require custom-coded implementations (Kéry and Royle, 2016, Kéry and Royle, 2020).

**S20: Borrow strength from other taxa in joint models.** Joint species distribution models [JSDM; (Ovaskainen et al., 2017; Pollock et al., 2014)] are another tool that can strengthen the inference and predictions of true species distributions from data affected by the sampling issues. In the case of gridded biodiversity data affected by sampling issues, the idea is that different species may be affected by the same sampling issues, and thus data on other species can improve estimation of the sampling (observation) process (Rapacciuolo et al., 2021). This is closely related to the abovementioned Hill's (2012) Frescalo algorithm, which also assumes a sampling process shared across species. Furthermore, thanks to niche conservatism, phylogenetically or functionally similar species may also have similar environmental requirements; modelling this in JSDMs can improve estimation of the ecological process (McInerny and Purves, 2011; Ovaskainen et al., 2017) even if the target species is undersampled. A related idea is to model ecological quantities at multiple taxonomic levels (Adjei et al., 2024). Examples of software packages available for JSDMs at large scales are HMSC (Tikhonov et al., 2020), sjSDM (Pichler and Hartig, 2021), or spOccupancy and spAbundance mentioned in S18 and S19 (Doser et al., 2022, Doser et al., 2024).

## 5 Problems of scale

Scale has several meanings. Here, we deal with two aspects of scale: (1) spatial and temporal *grain* or resolution, which is an average area of a grid cell or average temporal duration, also known as time span (Mellado-Mansilla et al., 2026), of an observation, and (2) spatial and temporal *extent*, which is the total area of a region covered by a gridded dataset, or the total temporal period covered by a series of gridded atlases in the same region. For instance, the last three editions of Czech bird atlases (Ortega-Solís et al., 2026; Šťastný et al., 2021) has spatial grain of 11.2 × 12 km, spatial extent is the whole Czech Republic, temporal grain is roughly 4 years (the average time covered by one edition), and a temporal extent of 30 years (from 1985 to 2015).

Most scaling problems emerge when comparing gridded datasets, or their editions, with different grains, or when generalizing results from a gridded dataset outside of its extent and grain. The problem of varying (or limited) extent is a problem of generality: the larger the spatial extent of a grid, the more general the pattern (or predictions, or inference) derived from that grid (Wiens, 1989). Hence, problems of extent are mostly solved by not generalizing (extrapolating) outside of the extent of the data. More fundamental are problems of grain (Fig. 4), since different grains reflect different ecological mechanisms. For this reason, we focus on problems and solutions related to spatial and temporal grain, not extent.

**Fig. 4:**
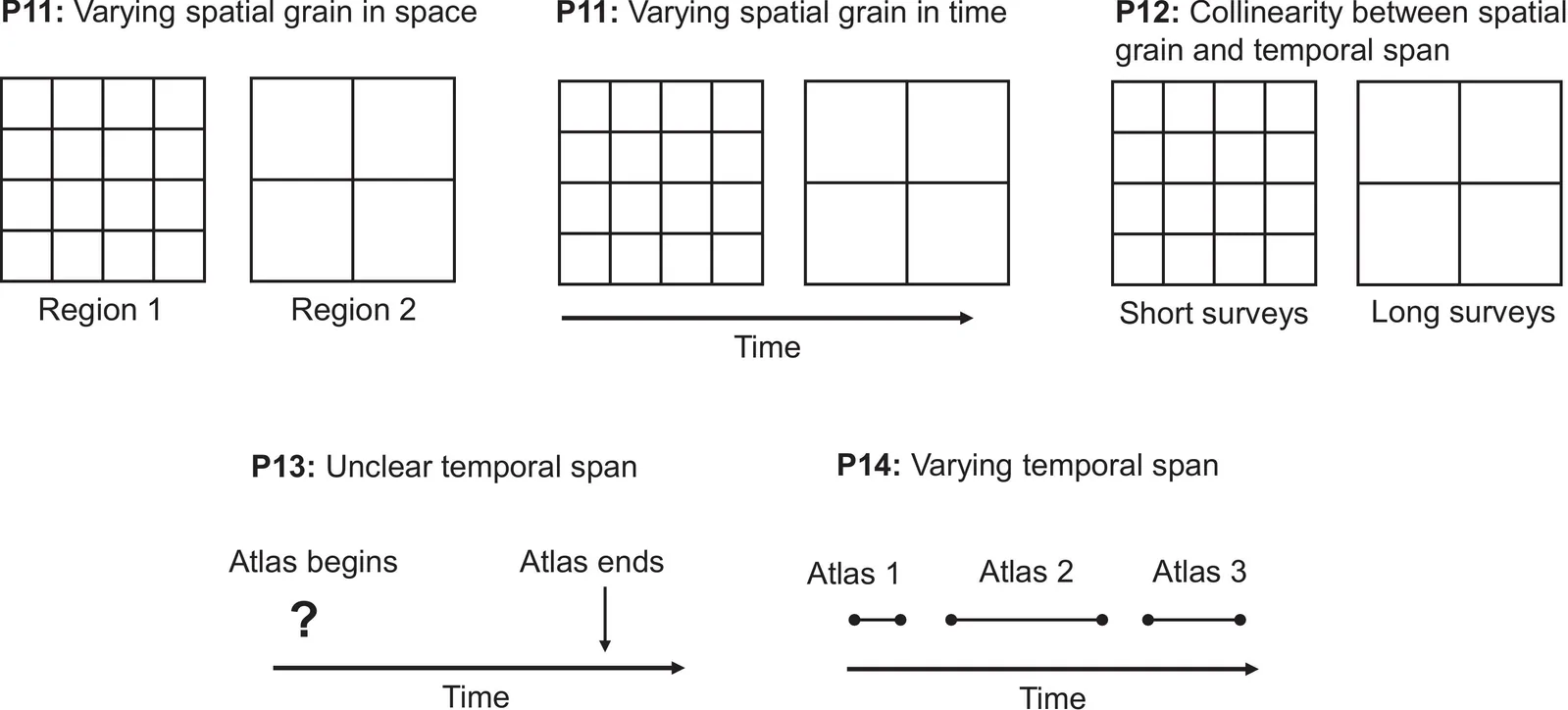
Common problems of gridded biodiversity data related to spatial and temporal scale.

### 5.1 Overview of problems of scale

**P11: Varying spatial grain in space or time.** Spatial grain of the grid affects species commonness and rarity (Hartley and Kunin, 2003), patterns of species diversity (Rahbek, 2005), turnover (Keil et al., 2012), aggregation (Soria et al., 2026), and also associations of these with the environment (Belmaker and Jetz, 2011). This also includes species-distribution models (SDM), which can lead to different inference and predictions depending on the grain of the data (Mertes and Jetz, 2018). The effect of temporal grain on patterns of species diversity and distribution is much less studied, but there are good theoretical and empirical reasons for temporal grain being similarly important as spatial grain (Mellado-Mansilla et al., 2026), mostly due to the species-time-area relationship (Adler et al., 2005). All this means that analyses of gridded data at one grain may be hard to compare to analyses using different grains.

**P12: Collinearity between spatial grain and time span.** We expect that the larger the spatial grain of gridded data, the longer the time span (Mellado-Mansilla et al., 2026) over which the data were aggregated (Fig. 4). Examples are European plant grids: The per-country data on European plant colonizations and extinctions (Essl et al., 2013) cover a broad time period of around 100 years, similarly to the 50 × 50 km European gridded plant atlas (Lahti and Lampinen, 1999) which covers a long period of time of more than 100 years, while smaller per-country gridded data [e.g. Stroh et al., 2023b or Klinkovská et al., 2024] cover intervals of decades. Although fine-grain data can be possible to aggregate to longer time periods, as far as we are aware this has not been done; conversely, we have not heard of a per-country system of regional species lists (as in Fig. 1a) that would cover only a short period of a year or two. When comparing analyses based on data with different spatial grains, one may then observe differences that may in fact be caused by different temporal spans.

**P13: Unclear temporal span.** Some gridded data are aggregations of all historical records that are available, with unspecified start year of the aggregation (Fig. 4). Examples are the per-country butterfly and plant species lists (Essl et al., 2013; Van Swaay and Warren, 1999) or the abovementioned European plant atlas (Stroh et al., 2023a). This can pose a challenge when assessing rates of change between two periods where the first period is undefined (Keil et al., 2018).

**P14: Varying temporal span.** Sometimes multiple editions of an atlas in a region can vary in the temporal extent that they cover. For instance, the four editions of the Czech bird atlas (Ortega-Solís et al., 2026; Šťastný et al., 2021) cover 5, 5, 3, and 4 years respectively. If used for assessments of temporal trends, a declining trend may be observed over the four editions simply because the first editions have longer time span.

### 5.2 Solutions to problems of scale

Below we offer two types of solutions to the problems of scale. The first ones (S21-S22) are mechanical adjustments of the grid aiming at making grains comparable, they are simple but may reduce sample size and fine-grain detail. The second type (S22-S25) are model based solutions which enable inference and predictions at different grains, even at grains not provided in the data, but can be challenging to implement.

**S21: Coarsen up the grid.** The simplest solution to the variation in spatial grain is to aggregate some or all grid cells to make them comparable with other coarse-grain data. This may also fix some of the geometrical and sampling issues (as described in the previous sections). Apart from the reduced sample size already mentioned in S6 and S14, the downside is that analyses of coarsened data will capture ecological processes that may differ from fine-grain processes. That does not mean they are incorrect, just different. For instance, species diversity and distributions at fine grains may be reflecting local stochasticity, while at coarse grains they will follow climatic gradients (Craven et al., 2020). Thus, predictions and inference based on models fitted to the coarsened data are not directly comparable with those from fine-grain original data.

**S22: Extend time span.** An analogical solution to the coarsening up the grid in space is merging multiple temporal replications to one, i.e. extending the time span. This can fix the issue of varying time span (Fig. 4), but comes at a cost of losing temporal detail, potentially also temporal replication, and it is only possible when many temporal replications are available, which can be a rare commodity. To see how extending the time span affects patterns and drivers of biodiversity see Mellado-Mansilla et al. (2026).

**S23: Downscale models.** A more advanced and challenging approach is to infer fine-grain distributions or diversity, or ecological processes, from the coarse grain data. This is typically done through hierarchical statistical models (see also S18) where likelihood of fine-grain distributions and parameters are conditional on coarse-grain data (Keil et al., 2013; Keil and Jetz, 2013), or when we assume a scale-free underlying point pattern intensity behind the grid (Isaac et al., 2019). The latter approach also allows integration of gridded data with other types of data for improved inference and predictions (Grattarola et al., 2023; Pagel et al., 2014). While conceptually elegant, the challenge of downscaling models is their computational cost and steep learning curve. On the other hand, if they work, these models make inference and predictions possible even at grains not represented by the data.

**S24: Use spatial grain or temporal span as a covariate.** When combining entire datasets (atlases) with different grid resolutions in a single analysis, we can include the grain as a covariate, or as an interaction term in statistical models, and we can then fix the grain to a constant when making inference or predictions (Keil and Chase, 2019; Midolo et al., 2025). In the temporal context, this has been done by Mellado-Mansilla et al. (2026). However, using grain as a covariate is only sensible if differences in grain between atlases are not collinear with other properties (such as geographic or environmental gradients). This solution has lower computational costs and is more user friendly than S23 proposed above.

**S25: Estimate scaling relationships**, not single-scale effects. Rather than a specific solution, this is more of a mindset to approaching biodiversity data, an approach where we give up the idea that there is a single “best” or “correct” relationship or mechanism behind biodiversity. Instead, relationships and mechanisms change continuously as we move from fine to coarse grains. We know that quantities such as species richness (Storch, 2016), their patterns such as latitudinal or altitudinal gradients (Rahbek, 2005), and their environmental drivers change with spatial grain (Belmaker and Jetz, 2011; Field et al., 2009; Keil and Chase, 2019). Let's call this relationship between grain and ecological quantity, or model coefficient, a scaling relationship. These scaling relationships put both the statistical inference and predictions in context of a given grain, enabling comparisons across datasets of different grains. Here, gridded biodiversity data are especially suitable for estimation of these scaling relationships, as they are spatially contiguous and can be coarsened to a set of nested scales. For examples of this practice see Hartley and Kunin (2003), Hurlbert and Jetz (2007) or Keil et al. (2013).

## 6 Perspectives and future directions

Fig. 5 provides a synthesis of the relationships between the problems and solutions reviewed in this paper. An important insight emerging from this synthesis is that the connection between problems and solutions is many-to-many: most challenges can be addressed by multiple approaches (especially challenges arising from sampling effort), and several approaches that we mention mitigate multiple challenges simultaneously, prominent examples being getting data on sampling effort or coarsening the grid. The latter ameliorates several sampling and scaling problems; however, here we also need caution as sample size may drop too much at too coarse resolutions. Nevertheless, this highlights opportunities to move beyond the individual treatment of geometry, sampling, and scale, towards a solutions for challenges from several domains at once. Hierarchical, integrated, joint, and cross-scale modelling approaches provide promising examples of this direction, but substantial opportunities remain for their development, particularly in terms of improving computing efficiency and user friendliness.

**Fig. 5:**
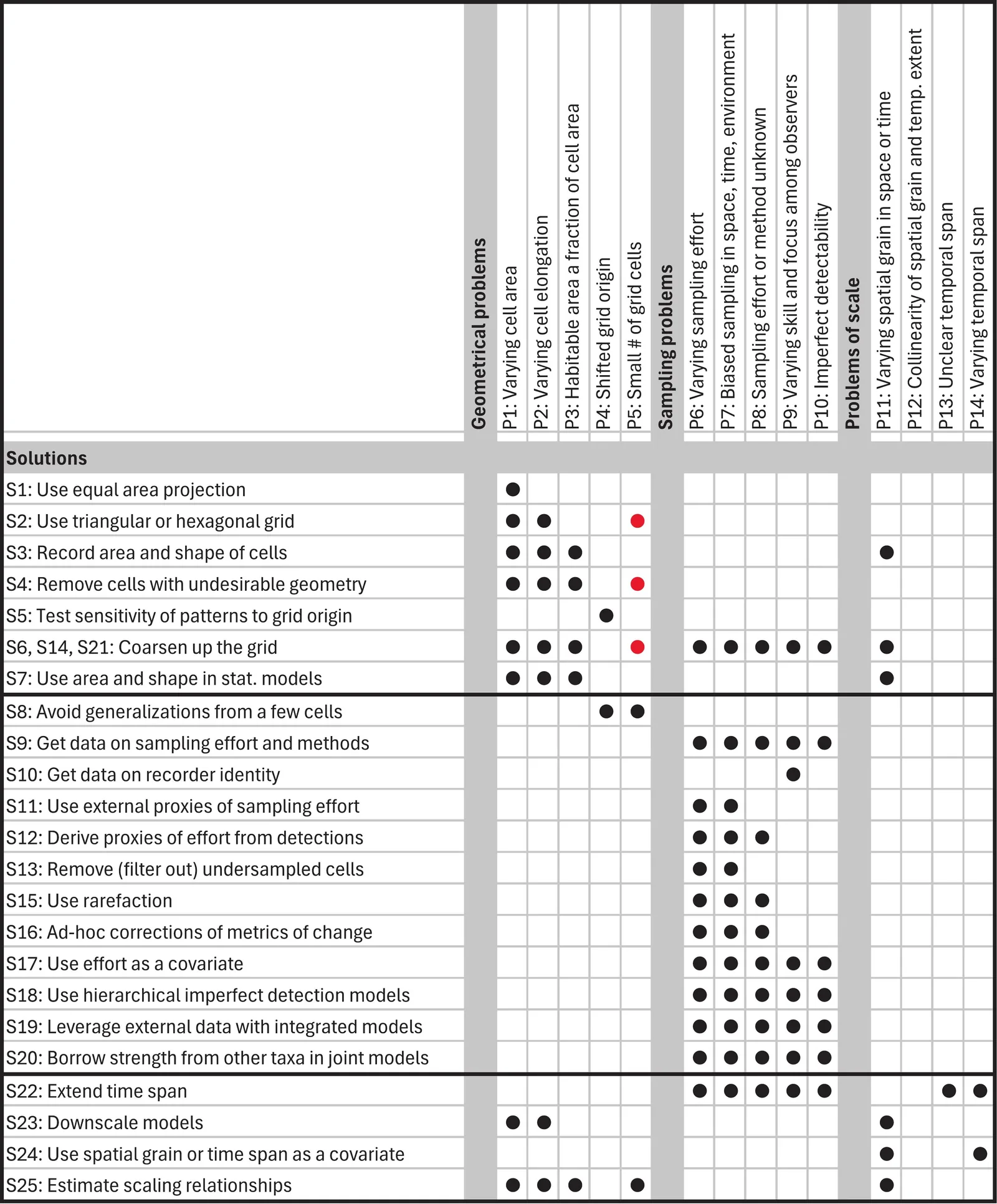
Connections between the problems and solutions. Black cells indicate that a solution (row) can help with a given problem (column). Red cells indicate that the solution would make the problem worse. The collinearity between spatial and temporal grain is the only problem for which we do not see a solution. (For interpretation of the references to colour in this figure legend, the reader is referred to the web version of this article.)

We also note that we have focused on problems that we know from our work, some of which are notorious (such as the sampling problems), whereas other problems only received limited attention (such as the problems of geometry and scale). However, there are whole families of important issues that we have omitted. Examples are problems of taxonomic standardization and taxonomic scale, problems of data ownership and licensing, or large-scale biases in geography and socio-economic history (e.g., most gridded data come from wealthy countries). Each of these would indeed deserve their own section, or even a paper.

## 7 Conclusion

This review demonstrates that the challenges associated with gridded biodiversity data are diverse but often manageable. Among the solutions discussed, several stand out because they are relatively straightforward to implement or can mitigate multiple problems at a time. These include: (1) Quantifying grid geometry (cell area, shape, and land proportion), (2) characterizing sampling effort through direct measurements or proxies (e.g. using the Frescalo algorithm of Hill (2012), or external variables such as accessibility or distance to institutions/cities), (3) choosing an appropriate spatial grain while considering the tradeoff between sample size and bias correction, and (4) applying modelling approaches that can account for geometry, sampling and scale, for example hierarchical occupancy models, integrated models, joint species distribution models, and cross-scale models.

At the same time, some challenges may be inherently difficult to overcome or have no solution at all. There are collinearities of all sorts (Dormann et al., 2013) and a lack of information about spatial or taxonomic variation in methods or abrupt changes in methodology between editions of atlases. In such cases, we can only hope to get some additional data or information. Furthermore, every solution reviewed here comes with assumptions and limitations, and hidden sources of variation and bias will inevitably remain.

Nevertheless, gridded biodiversity data remain among the most valuable resources available for understanding species distributions, diversity patterns, and their dynamics. By recognizing their limitations and applying appropriate solutions, researchers can substantially improve ecological inference and generate more reliable maps, predictions, and conservation insights.

## Declaration of generative AI and AI-assisted technologies

During the preparation of this work, the author(s) used AI tool Elicit to look for relevant papers (see the Literature processing section above). We did not use generative AI to write the manuscript; we wrote it manually. We also created all the figures manually.

## CRediT authorship contribution statement

**Petr Keil:** Writing – review & editing, Writing – original draft, Conceptualization. **Friederike Wölke:** Writing – review & editing, Writing – original draft, Conceptualization. **Gabriel Ortega-Solís:** Writing – review & editing, Writing – original draft, Conceptualization. **Florencia Grattarola:** Writing – review & editing, Writing – original draft, Conceptualization. **François Leroy:** Writing – review & editing, Writing – original draft, Conceptualization. **Florian Jansen:** Writing – review & editing. **Carmen Soria:** Writing – review & editing, Writing – original draft, Conceptualization.

## Declaration of competing interest

The authors declare the following financial interests/personal relationships which may be considered as potential competing interests: Petr Keil reports financial support was provided by European Research Council. Friederike Wölke reports financial support was provided by European Research Council. Gabriel Ortega reports financial support was provided by European Research Council. Florencia Grattarola reports financial support was provided by European Research Council. Francois Leroy reports financial support was provided by European Research Council. Carmen Soria reports financial support was provided by European Research Council. If there are other authors, they declare that they have no known competing financial interests or personal relationships that could have appeared to influence the work reported in this paper.

## Data Availability

No data or code were used in this article.
